# Silver nanoparticles elevate mutagenesis of eukaryotic genomes

**DOI:** 10.1093/g3journal/jkad008

**Published:** 2023-01-13

**Authors:** Kun Wu, Haichao Li, Yaohai Wang, Dan Liu, Hui Li, Yu Zhang, Michael Lynch, Hongan Long

**Affiliations:** KLMME, Institute of Evolution and Marine Biodiversity, Ocean University of China, Qingdao, Shandong Province 266003, China; Laboratory for Marine Biology and Biotechnology, Laoshan Laboratory, Qingdao, Shandong Province 266237, China; KLMME, Institute of Evolution and Marine Biodiversity, Ocean University of China, Qingdao, Shandong Province 266003, China; KLMME, Institute of Evolution and Marine Biodiversity, Ocean University of China, Qingdao, Shandong Province 266003, China; KLMME, Institute of Evolution and Marine Biodiversity, Ocean University of China, Qingdao, Shandong Province 266003, China; KLMME, Institute of Evolution and Marine Biodiversity, Ocean University of China, Qingdao, Shandong Province 266003, China; KLMME, Institute of Evolution and Marine Biodiversity, Ocean University of China, Qingdao, Shandong Province 266003, China; School of Mathematics Science, Ocean University of China, Qingdao, Shandong Province 266000, China; Biodesign Center for Mechanisms of Evolution, Arizona State University, Tempe, AZ 85281, USA; KLMME, Institute of Evolution and Marine Biodiversity, Ocean University of China, Qingdao, Shandong Province 266003, China; Laboratory for Marine Biology and Biotechnology, Laoshan Laboratory, Qingdao, Shandong Province 266237, China

**Keywords:** antimicrobial agents, mutagenic effects, mutation accumulation, fission yeast

## Abstract

Metal nanoparticles, especially silver, have been used in various medical scenarios, due to their excellent antimicrobial effects. Recent studies have shown that AgNPs do not exert mutagenic effects on target bacteria, but the degree to which they compromise eukaryotic genomes remains unclear. To study this, we evaluated the mutagenic effects of AgNPs on the fission yeast *Schizosaccharomyces pombe* ATCC-16979, of which ∼23% genes are homologous to human ones, at single-nucleotide resolution, and whole-genome scale by running 283 mutation accumulation lines for ∼260,000 cell divisions in total. We also explored the action and mutagenesis mechanisms using differential gene-expression analysis based on RNAseq. Upon AgNPs treatment, the genomic base-substitution mutation rate of *S. pombe* at four-fold degenerate sites increased by 3.46×, and small indels were prone to occur in genomic regions that are not simple sequence repeats. The G:C → T:A transversion rate was also significantly increased, likely mostly from oxidative damage. Thus, in addition to their antimicrobial potency, AgNPs might pose slight genotoxicity threats to eukaryotic and possibly human genomes, though at a low magnitude.

## Introduction

Over the past few decades, silver nanoparticles (AgNPs) have become one of the fastest-growing antimicrobial agents. For example, AgNPs can effectively inhibit over 650 pathogens, including viruses, bacteria, unicellular eukaryotes, and multicellular microorganisms (Jeong *et al*. 2005; Pal *et al*. 2007; Fayaz *et al*. 2010; Kim *et al*. 2012; Xiang *et al*. 2013; Juganson *et al*. 2017). Numerous reviews have summarized the bactericidal effects and diverse action mechanisms of AgNPs, including their effects on ribosomes or other functional proteins, and cell walls or membranes, as well as the formation of free radicals (Dakal *et al*. 2016; Zhang *et al*. 2016; Salleh *et al*. 2020). Our recent study demonstrated that AgNPs do not accelerate mutagenesis of target bacteria, but do trigger-resistance development (Wu *et al*. 2022). These observations indicate that AgNPs are promising in conquering the challenges from resistant pathogens, and provide the theoretical foundation for commercial products with metal nanoparticles.

With the growing popularity of nanotechnology, products with AgNPs have been applied in numerous medical supplies and personal items (El-Nour *et al*. 2010), and AgNPs production will reach 800 tons by 2025 (Mueller and Nowack 2008; Pulit-Prociak and Banach 2016). Meanwhile, it remains inconclusive whether AgNPs are mutagenic to human or non-pathogen eukaryotes subjects. Many studies suggest that AgNPs are of low toxicity, as evaluated by the changes of histological features, such as body weight and blood chemistry (Bergin *et al*. 2016; Garcia *et al*. 2016), but ignoring the side effects at the DNA level, i.e. genotoxicity to human or yeasts. Some previous studies indicate that AgNPs induce genotoxicity, by causing DNA oxidative damage and chromosomal breaks in mouse models—classical metrics for evaluating genotoxicity (Nallanthighal, Chan, Murray, *et al*. 2017; Nallanthighal, Chan, Bharali *et al*. 2017), while a thorough evaluation covering the whole-genome at the single-nucleotide level has not been performed.

MA experiment combined with whole-genome sequencing (MA/WGS) is currently the most precise method to estimate the genome-wide mutational features in evolutionary genetic studies (Bateman 1959; Mukai 1964; Halligan and Keightley 2009). During MA, dozens or hundreds of replicate lines from an ancestral individual are single-individual bottlenecked, usually for hundreds to thousands of generations, allowing for the accumulation of even highly deleterious mutations. Genetic drift from the single-individual bottlenecking is so strong that it can overcome the combined power of selection from both clonal expansion and treatments such as antibiotics, thus providing a mostly unbiased estimation of the genomic mutation rate and spectrum (Halligan and Keightley 2009; Long *et al*. 2016; Lynch *et al*. 2016). Recently, MA/WGS was successfully applied to evaluate genome-wide genotoxicity of many physicochemical factors at the single-nucleotide resolution, such as antibiotics, pH, herbicide, etc. (Long *et al*. 2016; Tincher *et al*. 2017; Strauss *et al*. 2017; Wu *et al*. 2022).

*Schizosaccharomyces pombe* (the fission yeast) is an excellent model organism for eukaryotic cell biology studies (Humphrey 2000; Davis and Smith 2001). About 23% of the fission yeast genes are homologous to humans, with highly conserved functions (Jin and Reed 2002; Kachroo *et al*. 2015). For example, 23 genes in the *S. pombe* genome are similar to human cancer-related genes involved in processes needed to maintain genomic stability, such as DNA damage, repair, checkpoint controls, and so on (Wood *et al*. 2002). In this study, we evaluated the mutagenic effects of AgNPs on eukaryotic genomes at single-nucleotide resolution and whole-genome level by running MA lines of *S. pombe* ATCC-16979 challenged by AgNPs. In addition, we inferred the action and mutagenesis mechanisms of AgNPs, using differential gene-expression analyses based on RNAseq.

## Materials and methods

### Strain, media, and preparation of AgNPs

*Schizosaccharomyces pombe* CBS-1042 (ATCC-16979) was ordered from American Type Culture Collection (ATCC). Yeast peptone dextrose (YPD) agar or broth (Solarbio, Cat No.: LA0220 and LA5010, respectively) was used for culturing during the minimum inhibitory concentration (MIC) testing, MA transferring, nucleic acid extraction, and cryopreservation. AgNPs were prepared following a previously described method by Hebeish *et al*. (2013). Briefly, 5 g of native maize starch was dissolved in 80 ml of distilled water at pH 12 by adding 1.5 g sodium hydroxide and stirred by a digital heating magnetic stirrer (MIULAB, TP-350S). After the starch was completely dissolved, we added 4.72 g AgNO_3_ into 20 ml distilled water, and then the silver nitrate solution to the reducing solution drop by drop. The mixture was incubated at 70°C for 1 h with continuously stirring, and cooled to 25°C. The products were rinsed with an equal volume of ethanol by centrifuging at max speed. AgNPs were characterized by following Wu *et al*. (2022): the absorption spectrum, size, surface traits, and zeta potential of the AgNPs were measured by a UNICO UV-2100 spectrophotometer, a Jeol (JEM-1200EX) transmission electron microscope, a MERLIN Compact-62-24 scanning electron microscope and a Malvern Panalytical™ Zetasizer Nano-ZS90 size analyzer, respectively. Finally, 10 mg/ml stock solutions of AgNPs were made by dissolving 0.4 g of AgNPs into 40 ml of nuclease-free water.

### MIC tests and the efficiency of plating

We determined the MIC following the method of Long *et al*. (2016). Cells were cultured to OD600 ∼1 on a shaker at 200 rpm and 26°C, then the culture was diluted to ∼1000 cells and incubated on YPD plates with a gradient of AgNPs concentrations (0, 20, 40, 60, 80, and 100 μg/ml). The survival curve for *S. pombe* was represented by the efficiency of plating (EOP), which was calculated by the *S. pombe* colony-forming units (CFU) of each group divided by the control CFU.

### MA experiments

Two groups of MA lines were initiated from a single ancestral colony and transferred by streaking onto YPD agar plates with or without 60 μg/ml AgNPs at 26°C. To avoid artificial selection for larger colonies, we only picked the last visible colony on the last streak. A total of 130 control MA lines were transferred onto YPD agar plates every 2 days; upon AgNPs treatment, 153 lines were transferred every 5 days, due to the growth inhibition by AgNPs. All MA lines underwent 50 transfers, experiencing approximately 20 and 17 cell divisions per transfer for the control and AgNPs treatment, i.e. ∼1,009 and 865 total cell divisions, respectively.

The number of cell divisions was estimated through CFU every 30 days. Specifically, 10 single colonies from different MA lines were chosen randomly and dug from the plate with sterilized toothpicks. Then, each colony was serially diluted in 1× PBS, plated, and the cell number *N* was counted. Assuming exponential growth in the colony, the division number (*T*) between transfers was calculated by *T* = log_2_*N*. The effective population size (*Ne*) was calculated by a harmonic mean method (Wahl and Gerrish 2001), where $Ne=\frac{T+1}{\sum_{i=0\;}^{T}\;\frac{1}{2^{i}}}$, where *T* is the number of divisions between transfers. All statistical analyses were done in R (v-4.2.1).

### Sequencing of MA lines and identification of mutations

Genomic DNA of *S. pombe* MA lines was extracted using an E.Z.N.A. Yeast DNA Kit (Omega Bio-tek, Cat No.: D3370-02). Then we prepared DNA libraries using a modified protocol for TruePrep DNA Library Prep Kit V2 for Illumina (TD501-1, Vazyme Biotech co., ltd) by following Li *et al*. (2019) and Illumina PE150 sequencing was performed on a NovaSeq 6000 sequencer of Berry Genomics, Beijing, China.

fastp (v-0.20.0) (Chen *et al*. 2018) was used to trim library adaptors and filter low-quality data of paired-end reads. Then we mapped the clean reads of MA lines to the reference genome of *S. pombe* (GWHBPBN00000000, China National Center for Bioinformation), using Burrows-Wheeler Aligner (v-0.7.17) MEM (Li and Durbin 2009). The HaplotypeCaller module of Genome Analysis Toolkit (GATK v-4.1.2) was used to call BPSs and indels using standard hard filters (McKenna *et al*. 2010; DePristo *et al*. 2011; Van der Auwera *et al*. 2013), and we validated candidate mutation sites by visualization in Integrative Genomics Viewer (Thorvaldsdottir *et al*. 2013).

For costs concern, we chose and constructed Illumina libraries for 80 and 90 MA lines of the control and the AgNPs treatment, respectively. After sequencing and mutation calling, if the two MA lines on the same Petri dish shared one mutation (we ran two MA lines on one YPD-agar Petri dish), then we removed the even-number line as the contaminated line. We removed three and one such contaminated lines in the control and the AgNPs treatments, respectively. We also set the minimum sequencing depth of coverage at 30*×*, and seven MA lines (four for the control and three for the AgNPs treatment) with lower coverage were filtered out. The mean mutation rate µ was then calculated by $\mu =\frac{m}{\sum_{1}^{n}\;N\times T},\;$ where *m* is the total number of mutations in all MA lines passing the filters, *n* is the number of MA lines, and *N* and *T* are the analyzed total sites and number of cell divisions in each MA line passaged, respectively.

### RNAseq

We streaked ancestor cells onto the YPD agar plates with or without AgNPs, with three replicates per group. After 48 and 120 h of incubation at 26°C for the control and the AgNPs treatment, respectively, cells on each plate were rinsed down with cold nuclease-free water and transferred into Eppendorf tubes on ice. Total RNA was then extracted using the E.Z.N.A. Yeast RNA Kit (Omega Bio-tek, Cat No.: R6870-01) and the concentration and purity of the RNA were measured with a Qubit 3.0 fluorometer and a micro-volume spectrophotometer instrument (Nano-300), respectively. We constructed RNA libraries using a NEBNext Ultra RNA Library Prep Kit and Illumina-PE150 sequencing was performed on a NovaSeq 6000 sequencer of Berry Genomics, Beijing, China.

### The differential genes expression, based on GO and KEGG analyses

Similar to mutation calling, low-quality RNAseq data and adaptors were filtered out with fastp (v-0.20.0). The clean reads were then mapped to the reference genome and converted to a SAM file by Hisat2 (v- 2.1.0) (Kim *et al*. 2019), and then sorted and converted to a bam file by SAMtools (v-1.9) (Danecek *et al*. 2021). Next, featureCounts (v-1.6.4) was used to generate a matrix of gene expressions (Liao *et al*. 2014) and DESeq2 was used for the differential genes expression (DGE) analysis with the parameter settings of padj > 0.01 and FoldChange < 2 (Love *et al*. 2014). The construction of the GO term database and GO enrichment analysis were performed using clusterProfiler (v-4.0) and filtered by adjustMethod ="BH", pvalueCutoff = 0.01, and qvalueCutoff = 0.01 (Wu *et al*. 2021). The plot of gene-wise dispersion is performed by the plotDispEsts() function of DESeq2 and the dispersion *α* is denoted by $\alpha =\frac{V-\mu }{\mu^{2}}$, where *V* and *μ* are the variance and mean of the number of reads mapped to each gene, respectively.

## Results

**Fig. 1.**
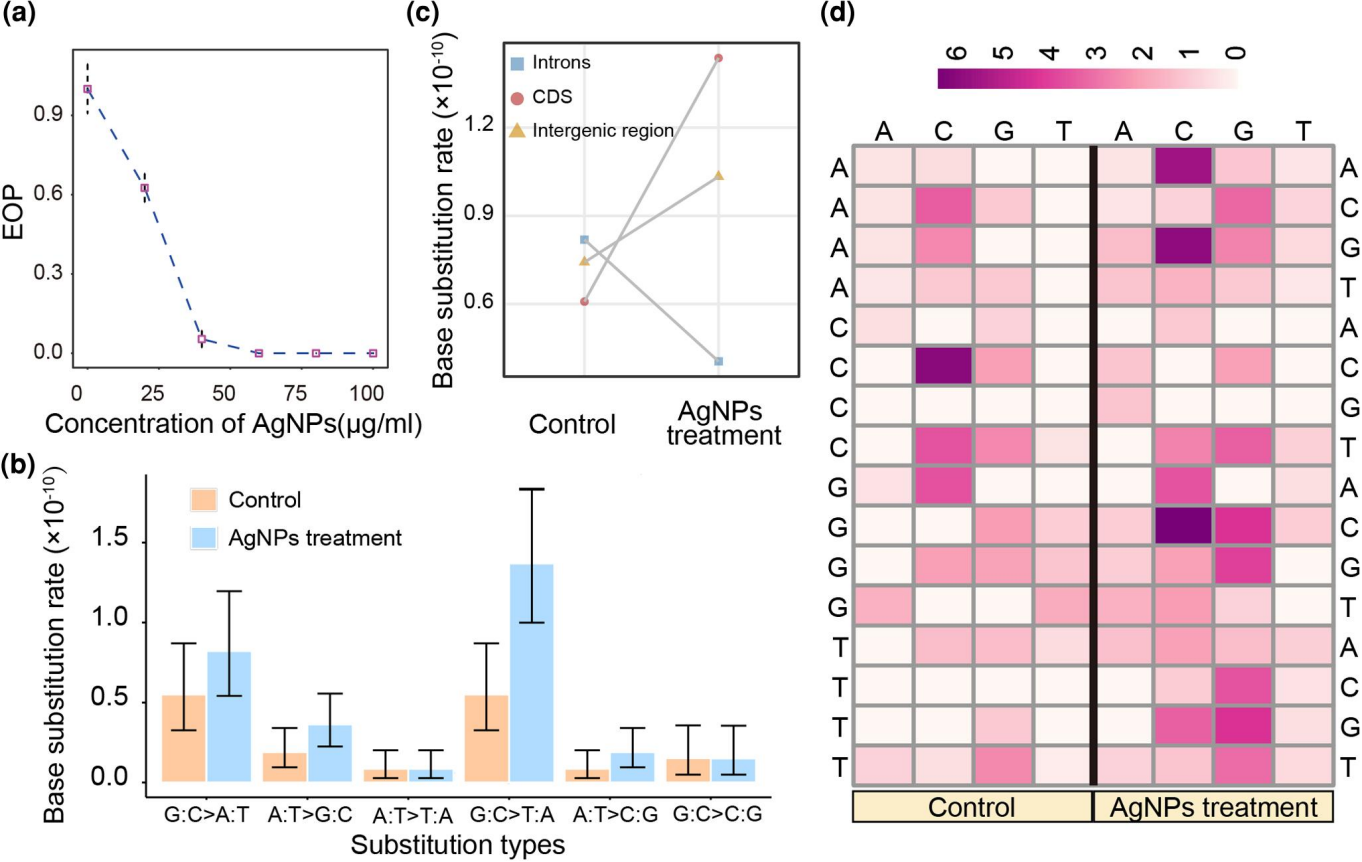
Survival curve and mutational profiles. a) The survival curve of *S. pombe* under the AgNPs treatment. b) Mutation spectra of the control and AgNP-treated MA lines. c) Mutation rates of different genomic regions in the control and the AgNPs treatment. d) Context-dependent mutation rates upon control (left) and AgNPs treatment (right). The heat map shows the mutation rates of each nucleotide context in the unit of 10^−10^ per site per cell division. 5' flanking, focal and 3' flanking nucleotides are shown at the left, top and right respectively.

We synthesized and characterized AgNPs with a mean diameter of ∼20 nm in the laboratory using a biological reduction method (Wu *et al*. 2022), modified from Hebeish *et al*. (2013), and then estimated the MIC of AgNPs on *S. pombe* ATCC-16979. Based on the EOP, the MIC is 60 μg/ml (Fig. 1a), which was used for both the MA and the RNAseq treatments. To evaluate the potential mutagenic effects of AgNPs on *S. pombe* ATCC-16979, we established 130 control and 153 treatment MA lines on YPD agar, with or without 60 μg/ml AgNPs, respectively. Each control or treatment line was single-colony streaked every 2 or 5 days, respectively, and experienced ∼1,009 and 865 cell divisions on average. After filtering out the MA lines with cross-contamination (four lines) or low-coverage <30*×* (seven lines), 73 control and 86 treated lines were used in the final analyses (Supplementary Tables 1–8). All the remaining MA lines were sequenced with the mean depth of coverage 49.44 (SD = 13.44) and mapping rate of 89.28% (SD = 4.63%), using Illumina PE150 mode.

Although single-cell bottlenecking minimized selection during the MA process, selection from both clonal expansion and AgNPs treatment might bias the accumulated mutations. To test this, we calculated the ratio of non-synonymous to synonymous substitutions of both the control and the AgNPs treatment lines. There was no significant difference between the control (there are 26 non-synonymous and 6 synonymous substitutions; *χ^2^* = 0.02, d.f. = 1, *P* = 0.89) and the AgNPs treatment (64 non-synonymous and 19 synonymous substitutions; *χ^2^* = 0.05, d.f. = 1, *P* = 0.82) in the ratios, by taking into account of the ratio of non-synonymous to synonymous sites (there are 6,074,090 non-synonymous and 1,641,376 synonymous sites in the genome) in *S. pombe*. We also estimated the effective population size (*N_e_*) during the MA process using a harmonic mean method (10.50 in the control and 9.00 in the treatment), suggesting that genetic drift dominated selection even upon AgNPs treatment. Moreover, we performed mutation enrichment analysis to identify genes with significantly elevated mutations—a sign of genes under positive selection, and only 0.15% and 0.04% genes exceeded the expectation in the control and the AgNPs treatment, respectively (Supplementary Tables 4 and 8). Thus, selection was minimal during AgNPs-treated MA, which could thus be used for evaluating mutagenic effects of AgNPs.

### AgNPs have mutagenic effects on base-pair substitutions but not indels in *s. pombe*

In total, 62 base-pair substitutions (BPS) were accumulated in the control lines, yielding a spontaneous BPS rate of 6.86 × 10^−11^ (95% Poisson CI: 5.26 × 10^−11^, 9.04 × 10^−11^) per nucleotide site per cell division, or 0.84 × 10^−3^ BPS per genome per cell division (95% Poisson CI: 0.65 × 10^−3^, 1.08 × 10^−3^; Supplementary Table 1). The estimated genomic mutation rate is ∼1/3 of the 2.42 × 10^−3^ (95% Poisson CI: 2.18 × 10^−3^, 2.67 × 10^−3^) reported in *S. pombe* ED668 (Farlow *et al*. 2015). The mutation rate is shaped by natural selection and random genetic drift (Lynch *et al*. 2016), and previous studies have suggested that mutation rates differ among strains (Ness *et al*. 2015; Pan *et al*. 2022). Thus, ATCC-16979 may be more efficient in DNA replication than other strains, which needs further exploration.

In the AgNPs treatment, the BPS mutation rate is 1.26 × 10^−10^ (95% CI: 1.04 × 10^−10^, 1.51 × 10^−10^; 114 BPSs accumulated in total) per nucleotide site per cell division (Fig. 1b; Supplementary Table 5), 1.84× higher than the control. We further calculated the mutation rate at the four-fold degenerate sites, at which BPSs usually do not result in changes at the amino acid level. The mutation rate at the 4-fold degenerate sites upon AgNPs treatment is even more strongly elevated than the control (3.46×; 4.95 × 10^−11^ vs 1.72 × 10^−10^; *χ^2^* = 4.5, d.f. = 1, *P* = 0.03), indicating that AgNPs are mutagenic to *S. pombe*. The 95% Poisson confidence intervals of the mutation rate at four-fold degenerate sites of the control are 1.35 × 10^−11^, 1.26 × 10^−10^; 9.38 × 10^−11^, 2.88 × 10^−10^ for the treatment. The overlapped CIs and the marginal *P* value probably result from low statistical power due to the small number of mutations accumulated. We also analyzed the small indels (insertions and deletions), and detected 34 and 23 small indels in the control and the AgNPs treatment MA lines, of which 100% and 65.22% are located in simple sequence repeats (SSRs) (Supplementary Tables 3 and 7). These yield indel rates of 3.76 × 10^−11^ (95% CI: 2.61 × 10^−11^, 5.26 × 10^−11^) and 2.54 × 10^−11^ (95% CI: 1.61 × 10^−11^, 3.81 × 10^−11^) in the control and the AgNPs treatment, respectively. In addition, indels are highly biased to insertions (insertion/deletion ratios 7.50 and 4.50 in the control and the AgNPs treatment, without significant difference; χ^2^ = 0.04, d.f. =1, *P* = 0.83). These observations demonstrate that AgNPs are mutagenic to the genome of *S. pombe* by elevating the BPS rate, but not the indel rate. Interestingly, AgNPs treatment significantly increases the proportion of indels that fall in genomic regions other than SSRs (0% in the control vs 34.79% in the AgNPs treatment; *χ^2^* = 7.76, d.f. = 1, *P* = 5.35 × 10^−3^). This pattern has also been seen in AgNPs-treated *E. coli* (35.71% vs 62.50%, based on data of the Supplementary Tables 5 and 9 of Wu *et al*. (2022)). We speculate that AgNPs alter the expression of some genes involved in DNA-repair systems (Long *et al*. 2018). In the presence of AgNPs, we do find two such genes showing differential expression: *pms2* (down-regulated, one member of the DNA mismatch repair) and *rad22* (down-regulated, involved in homologous recombination repair) (Supplementary Table 10). Whether and how such changes might coordinate to reduce indels located in SSR regions upon AgNPs treatment needs further exploration.

We also compared the mutation rates in coding sequences (CDS), introns, and intergenic regions (6.08 × 10^−11^, 8.19 × 10^−11^, and 7.42 × 10^−11^, respectively, in the control vs 4.05 × 10^−11^, 1.44 × 10^−10^, and 1.03 × 10^−10^ in the AgNPs treatment; Fig. 1c). The mutation rates of introns and intergenic regions of the control and the AgNPs treatment are not significantly different (*χ^2^* = 0.17, d.f. = 1, *P* = 0.68 for introns and *χ^2^* = 1.54, d.f. = 1, *P* = 0.21 for intergenic regions). But in the CDS, AgNPs treatment significantly increases the mutation rate by 1.76× (*χ^2^* = 16.80, d.f. = 1, *P* = 4.15 × 10^−5^), accounting for most of the genomic mutation rate increase. This could be due to transcription-associated mutagenesis, i.e. coding sequences are in single DNA strands after the unwinding for transcription, and prone to mutate (Jinks-Robertson and Bhagwat 2014). To further confirm this hypothesis, using the RNAseq datasets generated in this study, we compared the expression level (measured by FPKM, Fragments Per Kilobase of transcript per Million mapped reads) of mutated genes with that of the non-mutated ones. In both the control and the AgNPs treatment, the mean expression level of mutated genes is not significantly different from that of the non-mutated ones (for the control, *P* = 0.54; *P* = 0.37 for the AgNPs treatment), possibly due to the low statistical power conferred by the low number of mutations accumulated. More rigorous and direct tests are needed for this point in the future.

Mutation rates can be highly influenced by the flanking nucleotide context (Sung *et al*. 2015; Long *et al*. 2015). Among the 64 trinucleotide contexts in the control, the top three triplet-contexts with the highest mutation rate are 5'-C[N]C-3', 5'-C[N]T-3' and 5'-G[N]G-3', where the focal N is any nucleotide, consistent with that other organisms of which mutation rate is increased when the flanking nucleotides contain at least one G or C (Sung *et al*. 2015; Long *et al*. 2015) (Fig. 1d). Upon AgNPs treatment, the top three contexts ranked by mutation rates high to low are 5'-G[N]A-3', 5'-A[N]G-3' and 5'-T[N]G-3', which is consistent with the general pattern (Fig. 1d). The context-dependent mutation rate is thus governed by deeper molecular forces, such as base-stacking and van der Waal forces, which are not overwhelmed by exogenous environmental factors.

### AgNPs treatment alters the mutation spectrum

The ratios of transitions to transversions (Ts/Tv) are 0.88 and 0.73 in the control and the AgNPs treatment, respectively (Table 1; *χ^2^* = 0.19, d.f. = 1, *P* = 0.66), which is highly consistent with previous reports on mutation spectra of other yeast species or *S. pombe* strains (0.78–1.72) (Nguyen *et al*. 2020; Farlow *et al*. 2015). However, upon AgNPs treatments, the G:C→T:A transversion rate is particularly elevated (Fig. 1b), suggesting that AgNPs elevate oxidative damage, as most G:C→T:A transversions originate from 8-oxo-guanines in the DNA strands (Lynch 2007). The overrepresentation of G:C→T:A mutations in the treatment indicates that AgNPs may cause oxidative DNA damage, consistent with previous reports that AgNPs elevate reactive oxygen species (ROS) in the cell (Joshi *et al*. 2015; Dakal *et al*. 2016). The 8-oxo-guanines in the cellular nucleotide pool could also cause A:T→C:G mutations. Indeed, the mutation rate of A:T→C:G under the AgNPs treatment is also higher than that of the control, though not significantly, possibly caused by the low statistical power from the limited number of A:T→C:G mutations accumulated (Fig. 1b; Table 1; Supplementary Tables 1 and 5). These results infer that AgNPs can elevate the *S. pombe* mutation rate by inducing oxidative damage to nucleotides in both DNA strands and the nucleotide pool of the cell.

**Table 1.** Mutational features of the control and the AgNPs treatment. Count is the sum of BPSs observed; proportion represents the fractional representation of each type of BPSs to the total pool.

Categories	Control	AgNPs treatment
	Count/Proportion	Count/Proportion
**Intergenic regions**	27/0.44	38/0.33
**Coding regions**	31/0.50	74/0.65
ȃSynonymous	7/0.23	16/0.22
ȃNon-synonymous	24/0.77	58/0.78
**Introns**	4/0.06	2/0.02
**Transitions**	29/0.47	48/0.42
ȃA:T→G:C	11/0.38	21/0.44
ȃG:C→A:T	18/0.62	27/0.56
**Transversions**	33/0.53	66/0.58
ȃA:T→T:A	5/0.15	5/0.08
ȃA:T→C:G	5/0.15	11/0.17
ȃG:C→C:G	5/0.15	5/0.08
ȃG:C→T:A	18/0.55	45/0.68
**Insertions**	30/0.88	19/0.83
**Deletions**	4/0.12	4/0.17

### Mutagenesis mechanisms of AgNPs-treated cells revealed by RNAseq

**Fig. 2.**
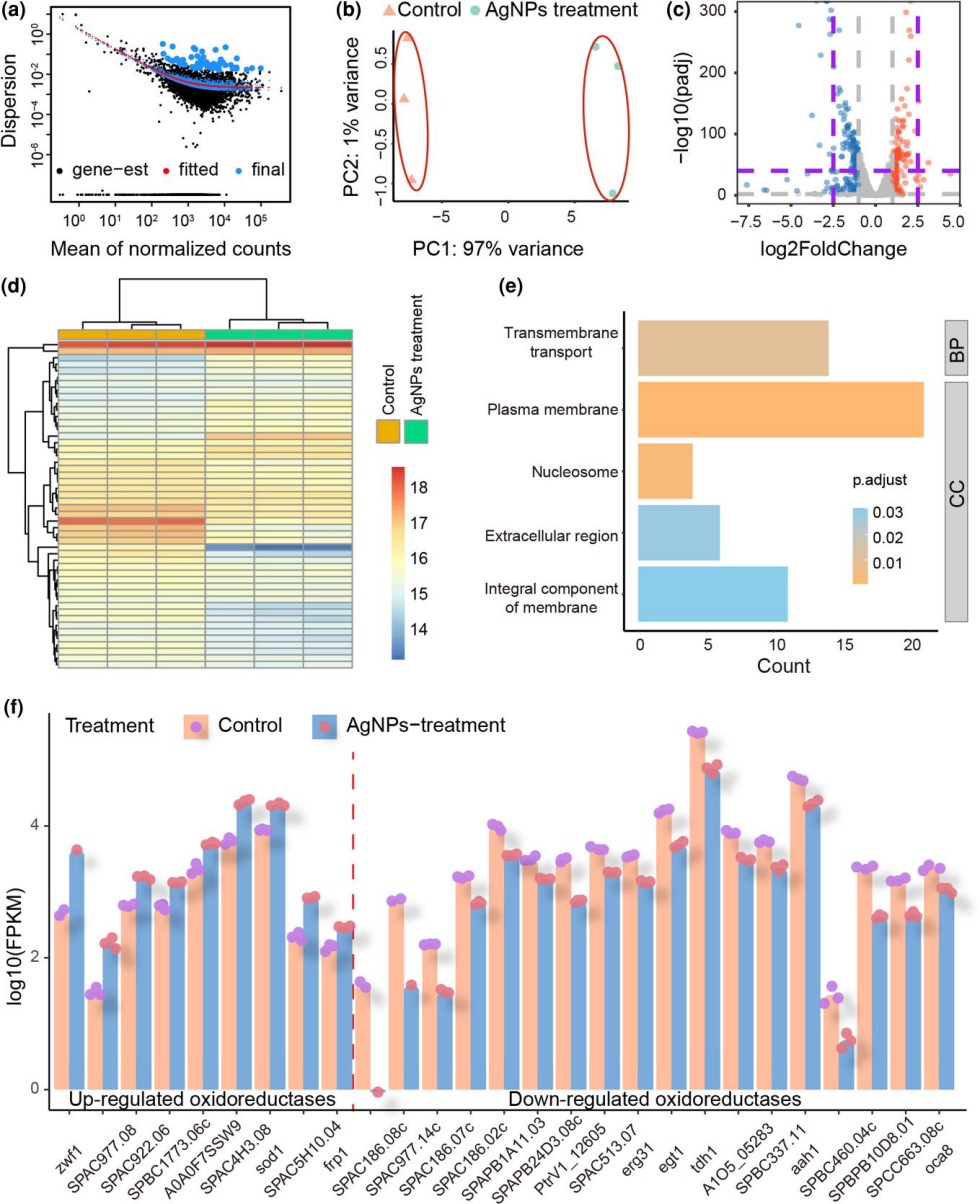
The differential gene expression of *S. pombe* upon AgNPs treatment. a) The estimation of gene-wise dispersions. b) The PCA plot based on gene expression. c) Differential gene expression upon AgNPs treatment vs control. Red, blue and grey dots represent significantly up-regulated, down-regulated and not significantly differentially expressed genes, respectively. The purple lines represent the threshold of extremely significant up or down-regulated genes (|log2FoldChange|>2.5 and −log10(padj) > 40; padj is adjusted *P* value using Benjamin-Hochberg correction). d) The heat map of top 50 differentially-expressed genes in the control and the AgNPs treatment after log2 transformation. e) Down-regulated genes from GO analysis under AgNPs treatment vs control. CC and BP represent cellular component and biological process, respectively. f) The FPKM of the up/down-regulated oxidoreductases after log10 transformation.

In order to further test this and other possible mutagenesis mechanisms, we extracted total RNA of the control and the AgNPs-treated (both with three replicates) lines of the ancestral strain, and performed RNAseq. With the RNAseq data, we performed Gene Ontology (GO) enrichment analyses to reveal the differential gene expression (DGE) (Fig. 2; Supplementary Table 9). After filtering out low-quality reads, a total of 124.87 and 137.62 million reads were obtained. The mean mapping rates for the control and the AgNPs treatment were 90.94% (SD = 0.26%) and 90.49% (SD = 0.61%), respectively. The RNAseq of different replicate lines were of high quality and repeatability, evaluated by dispersion, PCA, and heat-maps clustering analyses (Fig. 2, a and band d; Supplementary Table 9).

Compared with the control, there are 199 down-regulated and 155 up-regulated genes in the AgNPs treatment, including some with extreme significance and expression-level change (Fig. 2c). To elucidate the action mechanisms of AgNPs on *S. pombe*, we first parsed out the top 10 up/down-regulated genes (Supplementary Table 10; sorted by FoldChange from RNAseq analysis, *P* < 0.05). GO functional annotations on the down-regulated genes suggest that various catalytic reactions of metabolism dramatically decrease (Fig. 2e), such as hydrolase or nucleotidase (*SPAC186.09*, *SPBPB2B2.06c,* and *SPBPB2B2.05*), transport of nutrients or formation of biofilms (*SPBC8E4.01c*, *SPBC36.02c,* and *SPBC1348.06c*) and oxidoreductase (*SPAC186.08c* and *SPAC977.14c*) (Fig. 2f; Supplementary Table 10).

By contrast, there is no up-regulated PATHWAY in *S. pombe*. In the top 10 up-regulated GENES, we observe two oxidoreductases (*SPAC977.08* and *zwf1*; Fig. 2f). Whether these oxidoreductases are associated with AgNPs' induction of oxidative damage needs further exploration. There are also four up-regulated genes involved in metabolism or transcription: vitamin B6 transmembrane transporter (*SPCC576.17c*), hydrolase or lyase (*SPAPB1A11.02* and *SPBC8E4.05c*), and transcription factor (*SPAC2H10.01*). The vitamin B6 family, a coenzyme of diverse catalytic functions, mainly works in the metabolic conversion of amino acids (Ebadi *et al*. 1982). The up-regulated metabolism or transcription may reflect compensation for the inhibited energy metabolism, similar to bacteria challenged by AgNPs (Wu *et al*. 2022). Other differentially expressed genes are listed in Supplementary Table 10.

## Discussion

We recently demonstrated that AgNPs are not mutagenic to the model bacterium *Escherichia coli* (Wu *et al*. 2022). While the situation remains inconclusive for eukaryotic genomes, such possible genotoxicity might be harmful for humans and pose environmental risks via medical or daily consumables containing AgNPs. In this study, using MA/WGS and RNAseq, we find that AgNPs significantly increase the BPS mutation rate of the model yeast *S. pombe*, and change the mutation spectrum, particularly increasing the oxidative-damage-associated G:C→T:A transversions. Transcriptomic data also reveal the signs of AgNPs inducing ROS generation, e.g. up-regulation of certain oxidoreductases in the AgNPs-treated cells. In addition, our RNAseq data shows that AgNPs inhibit the growth of *S. pombe* primarily by disrupting the cell membrane or cell wall, as well as the transport of ions or substances (Fig. 2e; Supplementary Table 10). The application of AgNPs should thus be treated with caution and systematic studies on AgNPs action mechanisms are needed.

Even with highly similar research strategies (Wu *et al*. 2022), AgNPs exert different effects on prokaryotes (*E. coli*) and eukaryotes (*S. pombe*). Although the MICs of AgNPs on *E. coli* and *S. pombe* are the same (60 μg/ml), the median lethal concentration (LC50) of *S. pombe* is 40 μg/ml, lower than that of *E. coli* (Fig. 2c of Wu *et al*. (2022)), suggesting that *S. pombe* is more sensitive to AgNPs. Also, *E. coli* resists the influence of AgNPs through multiple up-regulated pathways to partially compensate for the decreased overall metabolism by AgNPs. By contrast, no up-regulated pathway was significantly enriched in *S. pombe*. In addition, AgNPs do not increase the genomic mutation rate of *E. coli*, and may even act as an anti-mutagenic agent (Wu *et al*. 2022), a strong contrast to AgNPs' mutagenic effects on the genome of *S. pombe*. These observations reveal that the effects of metal nanoparticles are highly dependent on the biological background.

ROSs can be induced by endogenous or exogenous factors, such as metabolites, ionizing radiation, chemical toxins, and so on (Lindahl 1993; Fry *et al*. 2005). ROSs can oxidize bases especially guanines, leading to aging and tumorigenesis (Karihtala and Soini 2007; Santos *et al*. 2013; Choromanska *et al*. 2021). Before this study, the oxidative damages on DNA caused by AgNPs were controversial, and the corresponding genotoxicity effects on the genome were also difficult to quantify. The up-regulation of diverse oxidoreductases implies the response to oxidative damages by *S. pombe* during AgNPs treatment (Fig. 2f; Supplementary Tables 10). To further understand the ROS damages, we checked the differential expression of the Sty1 and Pap1 pathway, which is critical for cell survival upon oxidative stress (Toone *et al*. 1998; Papadakis and Workman 2015). Unexpectedly, *sty1* and *pap1* are both down-regulated, even though the decreased expression of *pap1* is not significant, showing that AgNPs treatment did not trigger the expression of the oxidative-damage repair pathway (Supplementary Table 10). This may indicate that AgNPs also inhibit other functional proteins, since they are antimicrobial agents with multiple action mechanisms, and more complexity could be involved if the potency of AgNPs heavily depends on their size, shape, coated materials, and so on (Morones *et al*. 2005; Pal *et al*. 2007).

Taken together, this case study demonstrates that AgNPs elevate mutagenesis of eukaryotic genomes at single-nucleotide resolution, particularly by causing DNA lesions. More attention should thus be paid to side effects of commercial products containing metal nanoparticles, which could either increase the chance of the generation of drug-resistant eukaryotic pathogens or threaten human health by causing DNA damage.

## Supplementary Material

jkad008_Supplementary_Data

## Data Availability

All Illumina sequences used in this study were uploaded to NCBI BioProject PRJNA551791, Study No.: SRP222431. Supplemental material available at G3 online.
